# E-nside Off-the-Shelf Inner Branch Stent Graft: Technical Aspects of Planning and Implantation

**DOI:** 10.1177/15266028211047967

**Published:** 2021-09-27

**Authors:** Alexander Zimmermann, Anna-Leonie Menges, Zoran Rancic, Lorenz Meuli, Philip Dueppers, Benedikt Reutersberg

**Affiliations:** 1 Department of Vascular Surgery, University Hospital Zurich, Zurich, Switzerland

**Keywords:** thoracoabdominal aortic aneurysm, inner branch, off-the-shelf device, endograft, endovascular therapy

## Abstract

**Purpose:**

This article aims to present all aspects regarding patient selection, planning, and implantation technique for a new off-the-shelf pre-cannulated multi-inner branch stent graft. The stent graft comes in 4 different versions with proximal diameters of 33 and 38 mm and distal diameters of 26 and 30 mm. The 4 inner branches are located in the middle segment, which has a diameter of 24 mm.

**Technique:**

With inner branch technology, the field of application for the treatment of thoracoabdominal aortic aneurysms (TAAA) has been further extended. In addition to routine use in elective cases the pre-cannulation of the inner branches predisposes especially for emergencies. Pre-cannulation is intended to reduce the time to cannulation and the radiation dose. All steps of planning, stent-graft deployment, and cannulation of the inner branches are described in detail.

**Conclusion:**

The E-nside stent graft represents a promising new endovascular therapy in the treatment of acute and elective TAAA. By using inner branch technology, this endograft combines the advantages of fenestrated and branched stent grafts. Indication, planning, and implantation require experience in branched and fenestrated stent graft technology.

## Introduction

Endovascular therapy for aortic aneurysms has become a common alternative to open surgical therapy in recent years. Especially in the treatment of thoracoabdominal aortic aneurysms (TAAAs), open surgical therapy represents a high-risk intervention due to the risks of paraplegia, organ failure, or mortality.^1^ The introduction of fenestrated and branched stent grafts has significantly reduced these perioperative risks in the treatment of TAAA.^2^ The duration of stent graft production was also often a limiting factor, as urgent cases could not be treated due to the custom design of the grafts.

With the introduction of the first off-the-shelf multibranch endograft system in 2012, a large number of urgent TAAAs could be treated endovascularly.^3^ Only vascular morphology was a limiting factor for this prosthesis in a certain percentage of cases.^4^

Both the short- and long-term outcomes of these patients showed excellent results.^5–7^ Due to its outer branch design, the renovisceral segment should have a diameter of at least 30 mm according to the planning sheet, although diameters of 25 mm are possible in some cases with suitable morphology. Therefore, its use in pararenal or juxtarenal aortic pathologies is often not feasible.

With a new off-the-shelf inner branch stent prosthesis, it became possible to treat even these pathologies due to the 4 pre-cannulated inner branches. According to its instructions for use (IFU), renovisceral segments with a diameter of at least 24 mm can be treated. In addition, inner branches can combine the advantages of outer branches and fenestrations. While the advantages of outer branches are flexibility, improved sealing and fixation for bridging stents, and less critical positioning of the branches, the advantages of fenestrated prostheses are that they require less space (narrow aortas) and less aortic coverage.

This paper aims to describe all aspects of planning, stent graft deployment, and cannulation of the inner branches.

## Device Description

**Figure 1.**
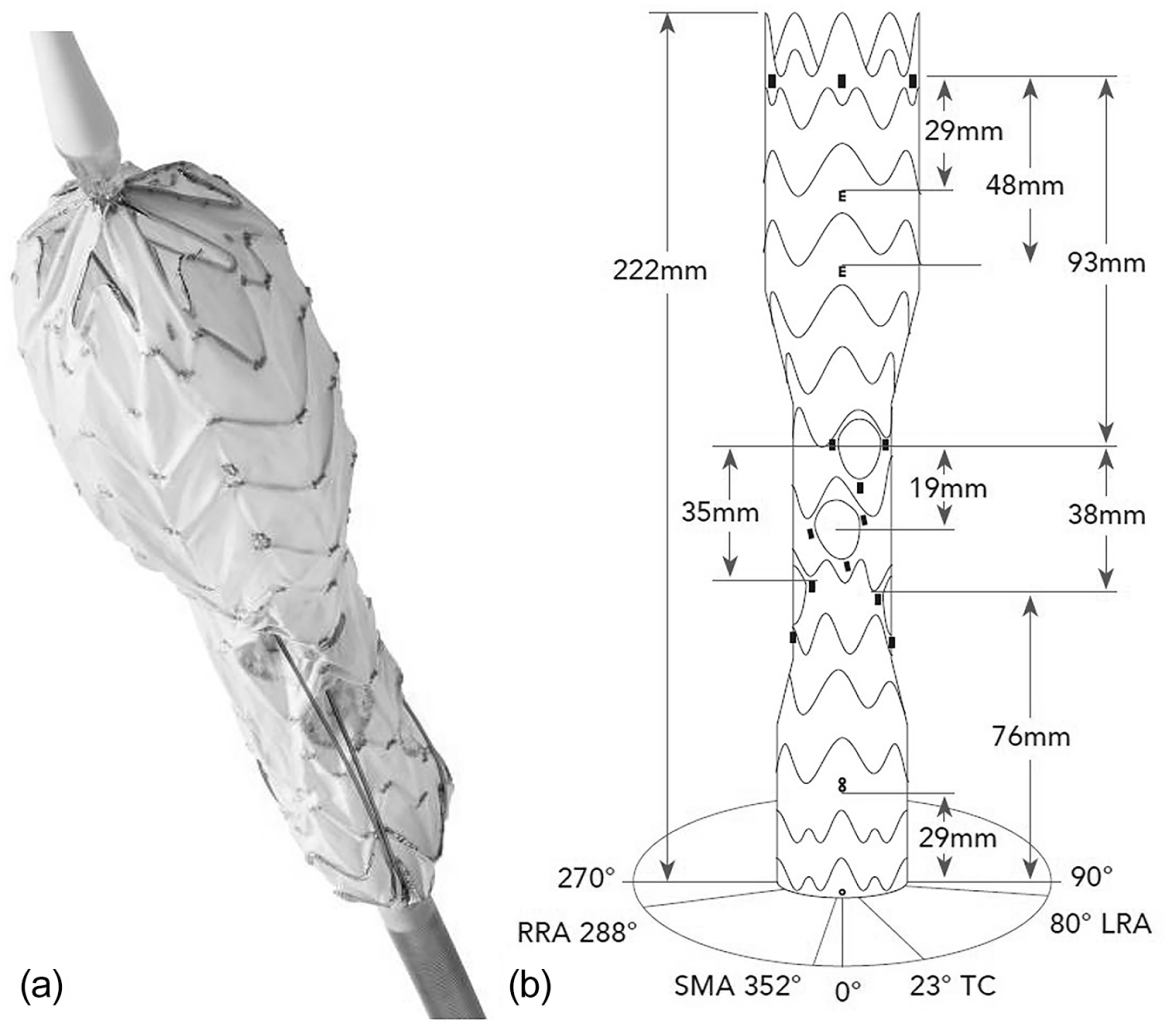
(a) The main body of the Cryolife/Jotec E-nside multi-inner branch stent graft with its preloaded polyimide tubes for easier cannulation. (b) Illustration of the different markers, distances, and orientation of the outlets of the inner branches. The proximal and distal stent graft portions have a peak-to-valley design, and the middle stent graft portion has a peak-to-peak stent design. RRA, right renal artery; SMA, superior mesenteric artery; TC, celiac trunk; LRA, left renal artery.

The E-nside prosthesis (JOTEC GmbH) is a multibranched endograft with 4 antegrade oriented inner branches for the renovisceral vessels (Figure 1). The prosthesis material is polyester, while the stents are electropolished nitinol. The prosthesis is available in 4 different versions with proximal diameters of 38 and 33 mm and distal diameters of 30 and 26 mm. The middle portion always measures 24 mm.

The 24 F delivery system comes with a nonhydrophilic atraumatic tip [8.2 mm outer diameter (OD)] and a hydrophilic outer sheath (90 cm working length, 8 mm OD). The stent graft is released by a so-called “squeeze to release mechanism,” where each click corresponds to a 4 mm step.

Different radiopaque markers are used for identification. A total of 5 tubular markers were used for the proximal and 3 ring markers for covering the distal end of stent graft. The inlet of each inner branch is marked with 1 ring marker, while the outlet is marked with 3 tubular markers. A total of 2 “E” markers display the maximum and minimum overlap for the proximal landing zone and provide rotational orientation [anterior/posterior (A/P) projection in the coronal plane]. An “8” marker (platinum–iridium) marks the distal overlapping line (30 mm from distal end of stent graft; Figure 1).

The proximal row of stents is fully covered and asymmetrically configured. This reduces the risk of wires/catheters being incarcerated in a bare stent. Additional advantages are the reduction of the windsock effect and the fact that catheters can be introduced before the proximal clasping is set free. The proximal and distal stent graft portions have a peak-to-valley configuration, giving the stent graft more flexibility. This results in better conformability to the aorta in the sealing zones. Furthermore, this design allows more contact points for a lower risk of infolding and a better distribution of the radial load onto the aortic wall. The middle stent graft portion, on the other hand, has asymmetric peak-to-peak designed stents to provide columnar support. The aim is that the design does not interfere with the inner branch cannulation (no edges) and that the patency of the inner branches is guaranteed throughout the cannulation phase (Figure 1).

The diameters of the celiac trunk (CT) and superior mesenteric artery (SMA) inner branches are 8 mm and those of the renal arteries are 6 mm, respectively. All inner branches have an antegrade orientation and a length of 20 mm. All 4 branches present with enlarged and oval-shaped outlets to allow for greater variability of the bridging stents. The design of the outlet of the inner branches results in flexibility and range in the axial plane of α=50° for the CT and SMA and β=70° for the renal arteries. In the sagittal plane, the degree of freedom is 0°<γ<45°.

All 4 inner branches were pre-cannulated with polyimide (PI) tubes with a length of 1465 mm, which extended to the proximal end of the stent graft. The inner diameter is 0.5 mm (0.018 in) and the OD is 0.7 mm (0.035 in). All tubes are loaded with a 0.018 in disposable transportation wire that has to be removed to use the pre-cannulation tubes.

## Anatomical Suitability Criteria

According to the IFU, the following requirements must be met:

- Adequate iliac/femoral access compatible with an 8.5 mm OD delivery system
- Angle ≤75°, 40 mm proximal to the CT
- Angle ≤30°, 20 mm distal to the lowest renal artery
- Length of proximal landing zone ≥30 mm
- Length of distal landing zone ≥30 mm
- No angulation in the proximal and/or distal landing zone
- No severe angulation in the thoracovisceral segment of the aorta ranging from 40 mm proximal to the CT to 20 mm distal to the lowest renal artery

According to the structural specifications of the E-nside endograft, the following requirements exist for the renovisceral vascular anatomy:

- The distance in height between the outlet of the inner branches of the stent graft and the ostia of the target vessels must be between 10 and 50 mm.
- Longitudinal angulation (sagittal plane) between the outlet of the inner branches and the ostia of the target vessels can be up to 45° for all visceral vessels.
- Lateral variability (axial plane) between the outlet of each inner branch and the ostium of its target visceral vessel can be up to 50° for CT and SMA and up to 70° for the renal arteries.
- All target vessels must be suitable for antegrade cannulation.

The current IFU requirements are subject to change as clinical experience with this novel stent graft increases.

## Technique

### Preoperative Planning

**Figure 2.**
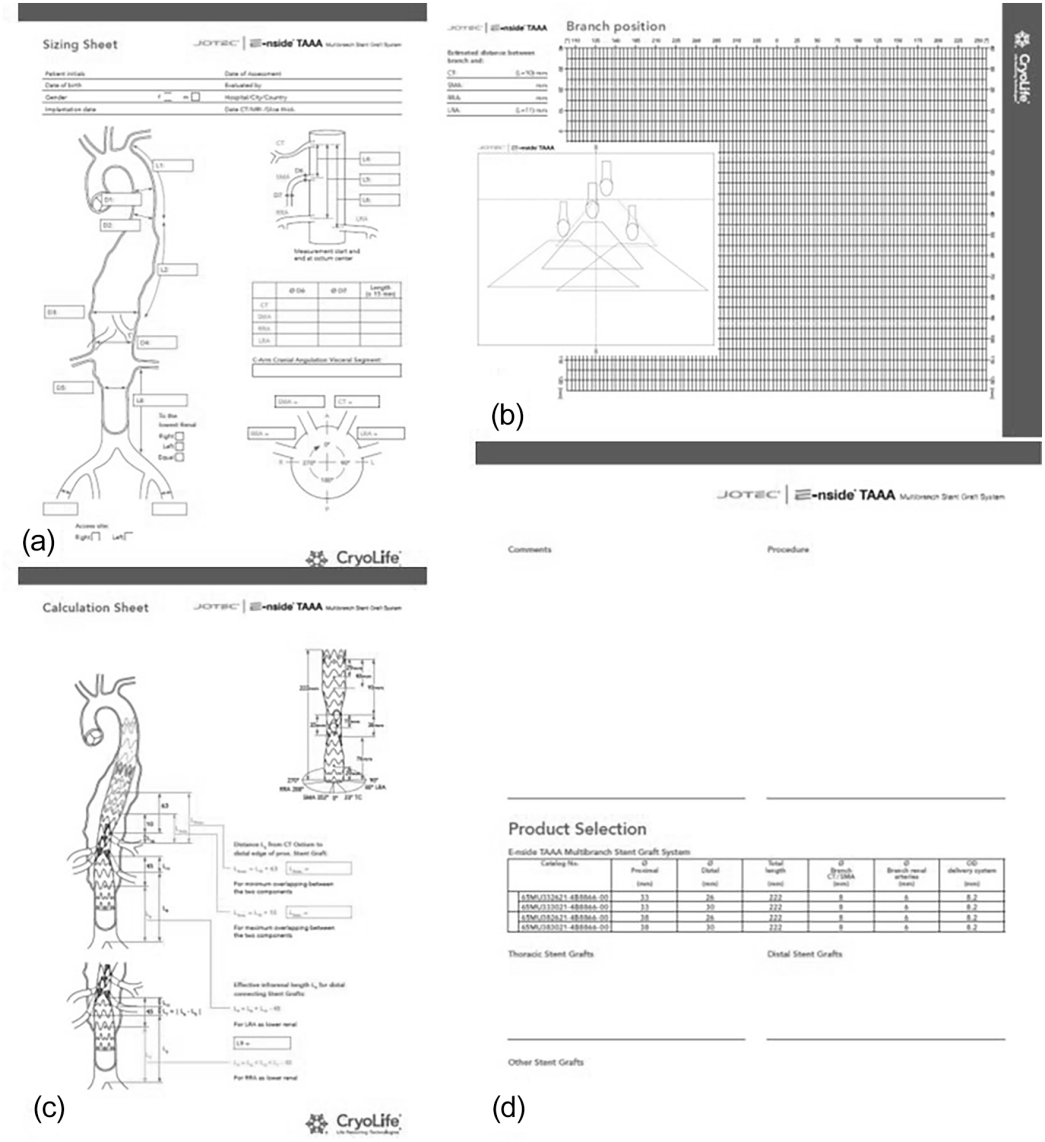
Different sheets for case planning. (a) Sizing sheet. (b) Branch position sheet. (c) Calculation Sheet. (d) Comments + Product selection sheet.

A total of 4 different paper sheets are available for planning and measuring (Figure 2). The first sheet (sizing sheet) provides an overview and the possibility to document the necessary measurements concerning lengths, diameters, and orientation of the vessel outlets (Figure 2a). All distances of the target vessels are measured from the center to the center of the vascular ostium. This requires a detailed examination and measurement of the images in multiplanar reconstructions.

After all measurements have been taken, they can be entered on the second sheet (branch position sheet) (Figure 2b). For target vessel assessment, CT serves as the starting point. This is plotted according to its degree on the zero line of the grid. The SMA and renal arteries are also plotted on the grid according to their orientation and distances from the CT. A transparent plastic sheet can then be used to determine anatomical suitability and the optimal position of the E-nside stent prosthesis. Each of the ostia of the target vessels must lie within the corresponding trapezoid of the respective inner branches. Accordingly, the center of the branch outlet should ideally be between 1 and 5 cm from the center of the ostium of the target vessel. By moving the template, the ideal position of the E-nside stent graft can be determined so that all 4 target vessels can be connected.

The third sheet (calculation sheet) can be used to determine the proximal and distal landing zones (Figure 2c). Here, it can be checked whether a sufficiently long proximal and distal overlap zone is present. If not, additional prostheses can be identified to provide sufficient sealing. In the proximal sealing zone, a suitable aortic segment must begin at least 20 mm and a maximum of 73 mm cranial to the ostium of the inner branch of the CT. In this regard, the IFU recommends an aortic diameter for the proximal landing zone between 27 and 30 mm for the 33 mm and 31 to 34 mm for the 38 mm E-nside stent graft. If an additional thoracic stent graft is necessary, the minimum and maximum distances from the CT ostium to the distal edge of the thoracic stent graft can be calculated. The same procedure is used for the distal landing zone. Here, the distance between the aortic bifurcation and the “8” marker, which marks the optimal onset of distal overlap, is determined. For the distal landing zone, an aortic diameter of 21 to 23 mm for the 26 mm and 24 to 27 mm for the distal 30 mm E-nside stent graft is recommended. For larger distal aortic diameters, the procedure must be completed with an aorto bi-iliac stent graft.

On the fourth sheet (comments + product selection sheet), comments on anatomic characteristics can be noted, and the corresponding product can be selected based on the calculated dimensions (Figure 2d).

### Implantation Technique

If thoracic aortic stent graft implantation is required, it should be performed first to ensure sufficient proximal stability. In principle, the implantation procedure should be from proximal to distal. Due to the length of the covered aorta, a spinal catheter should be considered in a 1-stage procedure to reduce the risk of spinal ischemia. For elective cases, so-called “Temporary Aneurysm Sac Perfusion” may be considered to achieve spinal vessel conditioning through residual perfusion of the aneurysm.^8^

Systemic full heparinization is performed according to body weight, with an activated clotting time of 250 to 300 seconds. The common femoral arteries are selected as access vessels via a surgical cut-down or percutaneous approach. A brachial or femoral approach can be selected for cannulating the inner branches. For the brachial approach, as reported in other studies, there are no differences in complication rates concerning side selection and should be adapted to the individual patient aortic arch configuration and conditions of the operating room/hybrid room.^9^ Retrograde cannulation and completion of the inner branches via femoral access is performed according to the technique published by Makaloski et al.^10^ This technique offers a lower risk of access site-related complications than the brachial approach and is the preferred technique at our institution.^10^

In the case of a previously implanted thoracic aortic stent graft, it is important to verify that its distal end is compatible in diameter (for at least 30 mm in length for overlap) with the proximal diameter of the selected E-nside stent graft. It must be positioned 20 mm (or more, according to the expected final position of the E-nside stent graft) proximal to the ostium of the CT. The E-nside delivery system is advanced in A/P projection via an extra stiff 0.035 in guidewire through the access vessel into the abdominal aorta. Tactile elevation of the gray handpiece of the delivery system must always point toward 12:00 and ensure correct alignment of the stent graft when it is advanced into the thoracoabdominal aorta. The 2 proximal “E” markers are used for fluoroscopic verification of the correct rotation of the stent graft. These markers have to face 12:00 in AP projection. Angiography is used to visualize the renovisceral segment. Still in A/P projection, alignment of the prosthesis in relation to the renal arteries is now performed under fluoroscopy control. For this purpose, the centers of the outlets of the renal inner branches are aligned according to the calculations on the “branch position sheet”. If necessary, a rotation adjustment must also be made according to the planning. After verifying the correct orientation and longitudinal position of the “E” markers and renal inner branches, the orange controller has to be brought on position “D”, and the deployment of the stent graft can be started by the “squeeze to release” mechanism. After deployment of the first few centimeters of the proximal stent graft portion, gentle adjustment of the stent graft position by slowly rotating the outer sheath and the black knurled cap with both hands can be performed if necessary until the intended orientation is confirmed once again. Proceed with the “squeeze to release” mechanism until the whole stent graft is deployed. To fully deploy and detach the stent graft from the delivery system, proximal tip capture must be released by rotating and pushing the knot at the distal end of the delivery system.

### Cannulation of the Branches

**Figure 3.**
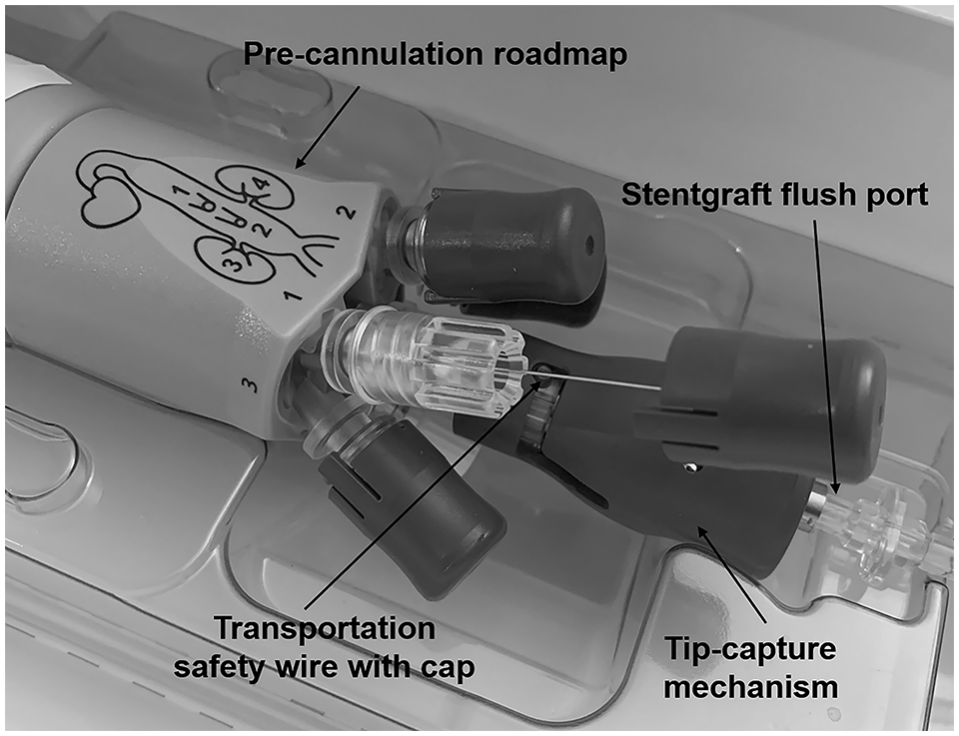
Handlebar with pre-cannulated polyimide tubes and safety wires. The celiac trunk is marked as “1”, the superior mesenteric artery is marked “2”, the right renal artery marked “3”, and finally the left renal artery is marked “4”. The tip capture at the distal end of the delivery system has to be rotated clockwise and pushed to release the proximal part of the E-nside stent graft.

**Figure 4.**
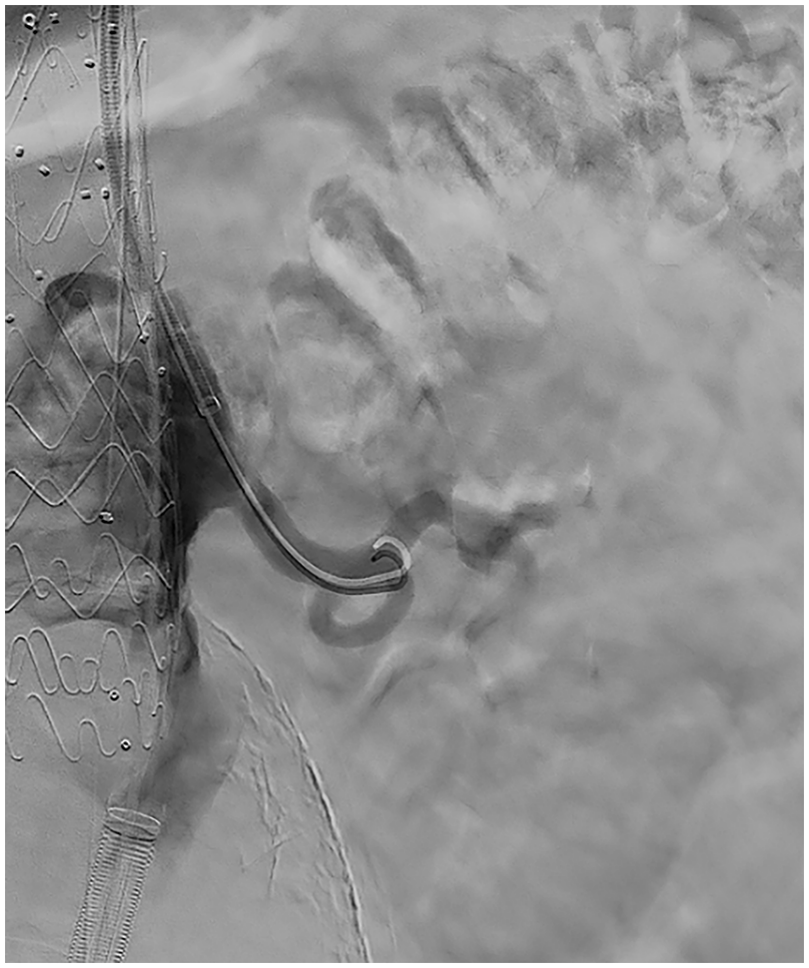
Intraoperative angiography of the left renal artery after transbrachial cannulation of the inner branch via the pre-cannulated wire with a sheath and unproblematic probing.

After full deployment, the cannulation process of the inner branches can be started. The sequence of the pre-cannulated PI tubes is shown on a sketch on the handlebar (Figure 3). The CT is marked as “1”, followed by SMA “2”, right renal “3”, and finally left renal artery “4”. Our preference is to usually start with the lower renal artery (e.g., “4”–“1”) in the case of transbrachial cannulation and with the CT (“1”–“4”) in case of cannulation from femoral to avoid compromising already completed branches during the probing process. After selecting the intended inner branch, the corresponding safe transportation wire must be removed. The PI tube has to be flushed with heparin–saline solution before a 0.018 in nonhydrophilic wire (Plywire Soft Tip 400 cm, OptiMed Medizinische Instrumente GmbH) can be advanced. The wire must be advanced over the proximal end of the E-nside stent graft to ensure that the wire tip does not remain in the PI tube during the subsequent snare maneuver. The wire can now be snared in the area of the thoracic aorta either via femoral or brachial access. After the through and through wire is established, the PI tube must be removed to ensure smooth advancement of the sheath. For the transbrachial approach, it is possible to insert a 12 F shuttle sheath with a femoro-brachial through and through wire (0.018 or 0.035 in) in advance for increased stability in the region of the aortic arch. Depending on the desired bridging stent graft, a 7 F (0.035 in through and through wire) or 8 F (0.018 in through and through wire) target sheath can be introduced via this shuttle sheath to probe the inner branches using the through and through 0.018 in wire. If a shuttle sheath is not desired, a 10 F sheath is inserted via transbrachial access and placed in the descending aorta. Either a flexible or a steerable 10 F sheath can be used from the femoral access site. A snare is inserted through the sheath, and the pre-cannulated inner branch wire is captured. After removal of the PI tube, the sheath is advanced into the inner branch via established through and through wire. Parallel to the through and through wire, a probing catheter can now be inserted into the sheath, and the target vessel is addressed (Figure 4). Before releasing the selected bridging stent graft, the through and through wire must be removed.

After all intended renovisceral vessels have been supplied with bridging stent grafts and connected to the inner branches, the orange lever at the handlebar of the delivery system must be brought into the “N” position. Now, the white “squeeze to release” handle must be held, and at the same time, the gray part of the delivery system must be slowly retracted under fluoroscopic control until the nose cone sits back onto the outer sheath. The entire delivery system can then be removed.

If necessary, the aorto bi-iliac E-tegra stent graft (JOTEC GmbH) with active fixation can be used for distal extension.

**Figure 5.**
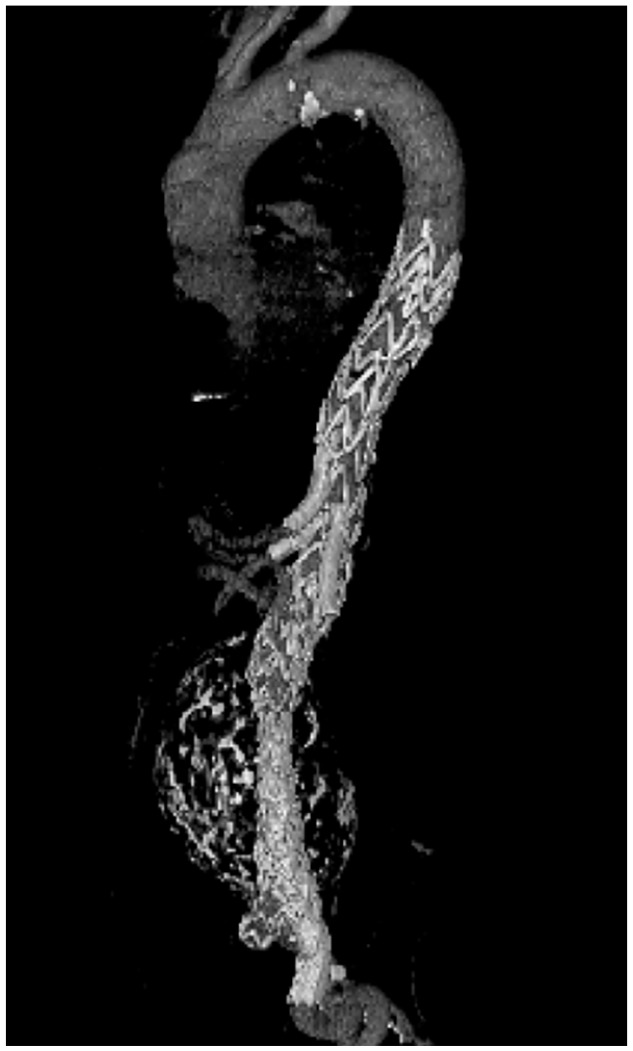
Postoperative volume rendering of a computed tomography angiography of a successfully implanted E-nside stent graft.

Final angiography is performed to assess the correct position of the stent prostheses and bridging stent grafts. If desired and technically possible, an on-table rotational or postoperative computed tomography scan can also be performed for better assessment (Figure 5).

## Discussion

The E-nside multi-inner branch stent prosthesis represents a new off-the-shelf device that attempts to combine the advantages of branched and fenestrated prostheses in the field of complex endovascular aortic repair. This is ensured by the inner branch design and the pre-cannulation technique.

The feasibility of this new device was tested in a retrospective analysis of Crawford I–IV TAAA and reported suitability of 43%. However, causes for exclusion mainly involved the iliac access vessels and proximal aortic diameters, while suitability regarding the renovisceral segment was given in 79% of patients.^11^ These results are comparable to the anatomical feasibility study by Bisdas et al^4^ for the Zenith t-Branch endograft (Cook Medical). It should be noted that these problems could be overcome by additional procedures, such as creation of an iliac conduit or additional proximal implantation of a tapered stent prosthesis; therefore, the actual clinical suitability should be higher.

The fact that good clinical results can be achieved with off-the-shelf multibranched endografts has already been demonstrated for the Zenith t-Branch. Early results showed both technical success and reintervention-free survival of 100% at a follow-up time of 5 months.^5^ In a recently published meta-analysis on the Zenith t-Branch prosthesis, good technical success rates and satisfactory mid-term results with a 30 day mortality of 5.8% were achieved in elective and urgent scenarios.^6^ Two studies demonstrated a 30 day mortality of 9%–14% for urgent and emergent treatment of TAAAs.^7,12^ This shows that these stent grafts are a safe alternative due to the off-the-shelf availability, thereby enable a rapid and precise therapy.

Due to the design of the E-nside inner branched stent graft and the minimum diameter of 24 mm in the renovisceral segment, it could also play a role in the treatment of urgent juxtarenal and pararenal aortic pathologies. Although an increased risk of paraplegia would have to be accepted due to the increased aortic coverage, the advantage lies in the less invasive nature of the treatment of these aneurysms, which can often only be treated with complex open surgery as an alternative in emergency situations.^13^ In addition, in very narrow anatomies, bridging stent grafts should be lined with a bare metal stent to avoid compression.

Although the inner branch design would offer the possibility of reducing the supraceliac coverage of the aorta and thus the risk of spinal ischemia, a comparison with the Zenith t-branch shows a similarly long distance from the beginning of the covering to the outlet of the CT branch (E-nside: 93 mm vs Zenith t-branch: 99 mm) because the E-nside is principally designed as a thoracoabdominal stent graft. However, the different proximal diameters of the stent graft, 33 and 38 mm, allow a wider range of thoracic aortas to serve as native landing zones, thus avoiding the need for an additional thoracic stent graft in some cases.

The restriction of the angle in the proximal (angle ≤75°, 40 mm proximal to the CT) and distal (angle ≤ 30°, 20 mm distal to the lowest renal artery) portion of the stent graft in the IFU is worth mentioning as it can have a limiting effect. This is mainly to avoid endoleaks (type I and III) in the landing or overlap zone, if proximal thoracic endovascular aortic repair (TEVAR) or distal extension is necessary. In addition, compression of the inner branch of the CT by excessive kinking in the proximal portion should be prevented.

In a first study, promising results with a thoracoabdominal stent prosthesis with an inner branch design could already be demonstrated. Advantages of the inner branch design were particularly noted for patients with complex/narrow aortas, postdissection TAAA, failed previous FEVAR cases, and for all those cases with an “awkward take-off of the viscerals” unsuitable for fenestrations or outer branches. It was found that inner branches that were not pre-cannulated were often difficult to cannulate.^14^ This is not the case with the E-nside stent graft, where all inner branches are pre-cannulated with a PI tube off-the-shelf. Due to pre-cannulation, the delivery system of the E-nside stent graft, similar to fenestrated stent grafts, must remain in the vessel until all branches and target arteries are completed. This may increase the risk of peripheral ischemia or even spinal cord ischemia. However, due to the high variability, pre-cannulation and low susceptibility to kinking of the inner branches, the time to cannulation of the target vessel can usually be kept very short.

One of the shortcomings of the current study is that at present, the first clinical results with the E-nside stent graft are still pending, and data on the durability and resistance of the inner branches, especially in anatomies with long bridging stent grafts, are lacking. Based on the first positive experiences in a premarket release and the already implanted numbers, these results should be available soon.

## Conclusion

The E-nside multi-inner branch endograft represents a new off-the-shelf device that attempts to combine the advantages of branched and fenestrated prostheses in the field of complex endovascular aortic repair. Although pre-cannulation of the inner branches simplifies implantation, this new aortic device involves a complex procedure, and advanced endovascular expertise is required to flatten the inevitable learning curve.
